# Mismatch Repair of DNA Replication Errors Contributes to Microevolution in the Pathogenic Fungus *Cryptococcus neoformans*

**DOI:** 10.1128/mBio.00595-17

**Published:** 2017-05-30

**Authors:** Kylie J. Boyce, Yina Wang, Surbhi Verma, Viplendra P. S. Shakya, Chaoyang Xue, Alexander Idnurm

**Affiliations:** a School of BioSciences, University of Melbourne, Parkville, Victoria, Australia; b Public Health Research Institute, Rutgers University, Newark, New Jersey, USA; c Department of Biochemistry, University of Utah School of Medicine, Salt Lake City, Utah, USA; Duke University Medical Center

**Keywords:** microevolution, mismatch repair, pathogens

## Abstract

The ability to adapt to a changing environment provides a selective advantage to microorganisms. In the case of many pathogens, a large change in their environment occurs when they move from a natural setting to a setting within a human host and then during the course of disease development to various locations within that host. Two clinical isolates of the human fungal pathogen *Cryptococcus neoformans* were identified from a collection of environmental and clinical strains that exhibited a mutator phenotype, which is a phenotype which provides the ability to change rapidly due to the accumulation of DNA mutations at high frequency. Whole-genome analysis of these strains revealed mutations in *MSH2* of the mismatch repair pathway, and complementation confirmed that these mutations are responsible for the mutator phenotype. Comparison of mutation frequencies in deletion strains of eight mismatch repair pathway genes in *C. neoformans* showed that the loss of three of them, *MSH2*, *MLH1*, and *PMS1*, results in an increase in mutation rates. Increased mutation rates enable rapid microevolution to occur in these strains, generating phenotypic variations in traits associated with the ability to grow *in vivo*, in addition to allowing rapid generation of resistance to antifungal agents. Mutation of *PMS1* reduced virulence, whereas mutation of *MSH2* or *MLH1* had no effect on the level of virulence. These findings thus support the hypothesis that this pathogenic fungus can take advantage of a mutator phenotype in order to cause disease but that it can do so only in specific pathways that lead to a mutator trait without a significant tradeoff in fitness.

## IMPORTANCE

Fungi account for a large number of infections that are extremely difficult to treat; superficial fungal infections affect approximately 1.7 billion (25%) of the general population worldwide, and systemic fungal diseases result in an unacceptably high mortality rate. How fungi adapt to their hosts is not fully understood. This research investigated the role of changes to DNA sequences in adaption to the host environment and the ability to cause disease in *Cryptococcus neoformans*, one of the world’s most common and most deadly fungal pathogens. The study results showed that microevolutionary rates are enhanced in either clinical isolates or in gene deletion strains with *msh2* mutations. This gene has similar functions in regulating the rapid emergence of antifungal drug resistance in a distant fungal relative of *C. neoformans*, the pathogen *Candida glabrata*. Thus, microevolution resulting from enhanced mutation rates may be a common contributor to fungal pathogenesis.

## INTRODUCTION

An essential prerequisite for all groups of microbial pathogens is the ability to change rapidly during infection to adapt to the hostile environment of the host. This adaption enables pathogens to avoid recognition or destruction by the host immune system, can result in latency and long-term pathogen persistence, and expedites the emergence of resistance to antimicrobial agents. One mechanism thought to account for the ability to change is the process of microevolution, represented by a change in allelic frequencies that occurs within a population over a short period of time. Microevolution during the course of infection can select for a small proportion of the microbe population that has gained novel traits that facilitate the ability to cause disease. These traits are generated by mutations in the microbe’s DNA sequence arising from changes at the chromosomal level, from errors in DNA replication, or from environmental damage. Mutations can be preexisting or can be rapidly acquired in response to the host environment in a process termed “adaptive evolution.”

Microevolution in fungal pathogens has previously been attributed to changes at the chromosomal level, with evidence of gain, loss, or recombination between chromosomes leading to resistance to antifungal agents (1–5). Upon exposure to the antifungal agent fluconazole, *Candida albicans* and *Cryptococcus neoformans* undergo a process termed heteroresistance in which rapid and yet reversible resistance is conferred by the development of one or more aneuploidies or large-scale chromosomal rearrangements (reviewed in reference 6). The most commonly occurring aneuploidy in *C. albicans* is an isochromosome of the left arm of chromosome 5, i(5L), which contains *TAC1* and *ERG11*, genes encoding the transcriptional activator of drug efflux genes and the target of fluconazole (7, 8). In *C. neoformans*, increasing concentrations of fluconazole result in duplication of chromosome 1, containing *ERG11* and the azole transporter *AFR1*, in all resistant strains, followed by successive duplication of chromosomes 4, 10, and 14 (9, 10). Cells return to normal ploidy when fluconazole is removed, presumably due to reduced fitness as evidenced by reduced proliferation and virulence *in vivo* (10). Interestingly, recent studies have also indicated that single-base-pair mutations may play an important role in the microevolution of pathogenic fungi during infection (11, 12). Genomic sequencing of *C. neoformans* isolates from a relapsed patient before and after antifungal treatment identified a chromosomal rearrangement and also an additional base pair mutation in the *AVC1* gene that controls several virulence phenotypes (11). In addition, high-frequency switching of *C. neoformans* between yeast cells to a pseudohyphal cellular morphology is governed by single-base-pair mutations in genes of the regulation of Ace2p activity and cellular morphogenesis (RAM) pathway (12). Importantly, mutation of *MSH2*, *MLH1*, and *PMS1* (three *C. neoformans* genes from the mismatch repair [MMR] pathway predicted to be required to repair damage to single bases arising from errors in DNA replication) resulted in increased proliferation in a lung assay of cryptococcosis (13). This suggests that single-base mutations arising from errors in DNA replication can generate novel traits that facilitate the ability to cause disease and therefore provide an avenue for the pathogen to undergo microevolution during infection.

Errors generated during DNA replication are corrected by the action of two sequential repair systems, namely, the DNA polymerase 3′–5′ exonuclease activity system and the mismatch repair (MMR) system. Possession of sequential repair mechanisms enables a multiplicative effect, increasing the fidelity of DNA replication by a factor of 10^4^ to 10^5^. In bacteria, replication errors are corrected by the 3′–5′ editing exonuclease activity of the ε subunit of DNA polymerase III and the *mutHLS* mismatch repair system (reviewed in reference 14). A MutS homodimer binds the mismatch and is detected by a MutL homodimer, which initiates removal by MutH nicking of the nascent strand. This system is structurally and functionally conserved in eukaryotes. In *Saccharomyces cerevisiae*, for instance, the Pol2 (ε) and Pol3 (∂) DNA polymerases, which primarily replicate the leading and lagging strands, respectively, also have 3′–5′ exonuclease activity. Subsequent repair is then performed by an analogous MMR pathway. The *S. cerevisiae* genome encodes six MutS homologues (*MSH1* to *MSH6*) and four MutL homologues (*MLH1* to *MLH3* and *PMS1*); however, only *MSH2*, *MSH3*, *MSH6*, *MLH1*, and *PMS1* are required for mismatch repair of nuclear DNA, with *MSH3* and *MSH6* playing partially redundant roles (reviewed in reference 15). *MSH1* is required for repair of mitochondrial DNA, whereas *MSH4*, *MSH5*, *MLH2*, and *MLH3* are involved in the meiotic recombination processes (15). In eukaryotes, the MutS homodimer found in prokaryotes is replaced with heterodimers referred to as MutSα (Msh2 and Msh6) and MutSβ (Msh2 and Msh3), which are redundant with respect to repairing small indels but are specialized with respect to removal of specific base-base mismatches and large insertion-deletion loops, respectively. MutSα/β is tethered to the proliferating cell nuclear antigen (PCNA), a DNA clamp that acts as a processivity factor for Pol3 (DNA polymerase ∂), and, after mismatch recognition, a MutL heterodimer referred to as MutLα (comprising Mlh1 and Pms1 [Mlh1-Pms1]) interacts to form a ternary complex that leads to nicks on the new strand and subsequent strand removal and resynthesis. The *S. cerevisiae MSH2*, *MLH1*, and *PMS1* MMR mutants exhibit a mutator phenotype in which the rate of spontaneous mutation is elevated (15).

This study investigated the contribution of single-base-pair mutations arising from errors in DNA replication to microevolution in a causative agent of cryptococcosis, *C. neoformans*. Cryptococcosis, caused by *C. neoformans* or *C. gattii*, is a significant fungal disease worldwide, with high incidence and morbidity. An estimated 624,000 deaths were once attributed to cryptococcosis each year, a rate higher that attributed to tuberculosis (16). The direction that this research took is presented as follows. Analysis of a collection of environmental and clinical *C. neoformans* isolates revealed that a number of isolates possessed a mutator phenotype and the ability to change rapidly due to the accumulation of a high frequency of DNA mutations. Whole-genome sequence analysis of two of these mutator strains revealed mutations in *MSH2* of the mismatch repair pathway. Deletion of the MMR components encoded by *MSH2*, *MLH1*, and *PMS1* in *C. neoformans* resulted in an elevated mutation rate due to an increase in mutations in homopolymeric tracts and transitions, specifically, G to A, suggesting that MMR in *C. neoformans* repairs errors arising from both replication and oxidative damage. We show that the elevated mutation rate and the types of mutations generated in the MMR mutants are not detrimental to growth and result in only minor sensitivity to stress while enabling rapid microevolution to occur. This microevolution introduces phenotypic variations in traits associated with the ability to grow *in vivo*, in addition to allowing the rapid generation of antifungal resistance. Interestingly, the elevated mutation rate of the MMR mutants was not detrimental to *C. neoformans in vivo*, with no decrease in colonization or virulence observed in *msh2*Δ and *mlh1*Δ mutants in a murine infection model. This suggests that *C. neoformans* isolates possessing mutations in MMR components have an opportunity for enhanced adaptive evolution during infection.

## RESULTS

### *Cryptococcus neoformans* clinical isolates exhibit a mutator phenotype.

**FIG 1:**
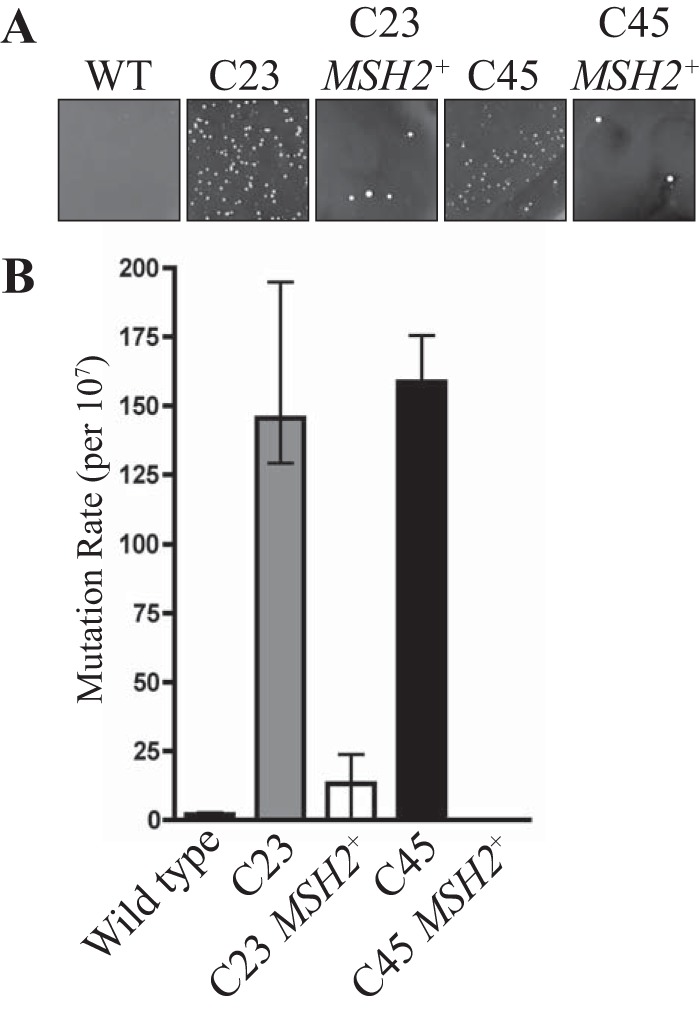
Two clinical isolates of *C. neoformans* display an elevated mutation rate due to mutations in the *MSH2* gene. (A) Spontaneous 5-FOA-resistant colonies in a standard wild-type (KN99α) isolate, the C23 and C45 clinical isolates, and the C23 and C45 isolates with a wild-type copy of *MSH2* introduced at an ectopic location. (B) Quantitative assessment of mutation rates in the five strains using fluctuation analysis of the spontaneous resistance to 5-FOA. The Lea-Coulson method of the median was used to estimate the number of mutations from the observed values of mutants from 20 independent parallel cultures.

Pathogens can retain a larger-than-expected proportion of mutators, i.e., cells with a greater-than-normal mutation frequency, in their populations (17–19). In bacteria, mutators found in nature carry defects in a component of the MMR (20). In addition, 55% of clinical isolates of the fungus *Candida glabrata* contain a mutation in *MSH2* (21). To assess if this phenomenon also occurs in *C. neoformans*, a collection of 11 clinical and 11 environmental strains of *C. neoformans* (var. *grubii*, serotype A) from the study described in reference 22 was examined for their mutation rates. This collection of strains is currently the best comparator set, in that the virulence of the strains has been tested, showing a correlation between virulence and clinical origin (22). Mutation rates in these strains were qualitatively assessed by observing the generation of spontaneously resistant 5-fluoroorotic acid (5-FOA) colonies (Materials and Methods). 5-FOA inhibits the growth of wild-type strains as 5-FOA is converted by orotine-5´-monophosphate decarboxylase to 5-fluorouracil (5-FU), which is toxic. Using a high-throughput rapid assay for the emergence of resistance, no differences in mutations rates were seen in comparisons of the 11 environmental isolates to each other or to a laboratory wild-type strain (KN99α). In contrast, 2 of the 11 clinical isolates, C23 and C45, had an increase in the number of spontaneously resistant 5-FOA colonies indicative of an increased mutation rate (Fig. 1A). C23 originated from bronchoalveolar lavage fluid of a lung transplant patient and C45 from the cerebrospinal fluid of an asthma patient (22). The increased mutation rate was quantitatively assessed using fluctuation analysis (Materials and Methods). This fluctuation analysis involves the inoculation of 20 parallel cultures (in nonselective medium) with a small number of cells (1 × 10^5^ cells) that are grown to saturation followed by plating onto selective media to obtain the number of mutants (Materials and Methods). The Lea-Coulson method of the median (23) was used with FALCOR software to estimate the number of mutations, from the observed values of mutants, across the 20 independent parallel cultures. The C23 and C45 clinical isolates showed mutation frequencies approximately 200-fold higher than those seen with the standard KN99α wild type (Fig. 1B).

To determine the molecular basis of the increased mutation rate, the genomes of the C23 and C45 isolates were analyzed by next-generation sequencing (Materials and Methods). Reads were aligned to the *C. neoformans* H99 genome sequence and single nucleotide polymorphisms (SNPs) and sequence variations identified (Materials and Methods). A total of 427 SNPs and sequence variations were identified in the C23 genome sequence. We hypothesized that an increased mutation rate would likely be a consequence of a mutation in a DNA repair gene, so those variants located within the coding region of the 57 genes predicted to play a role in DNA repair in *C. neoformans* were identified. *MSH2*, which is 1 of the 57 DNA repair genes in the C23 genome sequence, contained a sequence variation. *MSH2* encodes a MutS protein required for mismatch repair of nuclear DNA. The genome of isolate C23 contained a single-base deletion at +1689 which results in deletion of amino acids 379 to 397 and 3 missense mutations (V378N, S398L, and K399E). This region of the 965-amino-acid-long predicted protein (position 378 to position 399) contains 7 amino acids (R380, Q381, L388, P392, D393, R396, and K399) which are highly conserved from *S. cerevisiae* to humans. Furthermore, the mutational profile of the C23 genome resembles that of a *S. cerevisiae MSH2* mutant (24, 25). The majority of variations in C23 are SNPs arising from transition (42.9%) and transversion (22.9%) mutations and single-nucleotide deletions or insertions occurring within homopolymeric tracts (22.5%).

In contrast to that of isolate C23, the genome of isolate C45 showed an extremely high level of genetic heterogeneity compared to the genome of H99, with a predicted 269,177 SNPs and genome variations. A total of 32 SNPs were present within *MSH2* in isolate C45, with 21 within the coding sequence. Most of these SNPs resulted in silent mutations; however, 6 (V167A, T182I, D238V, L436P, S885F, and N863D) resulted in missense mutations. None of the missense mutations were in highly conserved amino acids; however, the T182I substitution is located in an equivalent position that, when mutated in *S. cerevisiae* (L183P), resulted in a 220-fold induction of mutation rate due to a lack of structural integrity (24).

To test if the increased mutation rate in the C23 and C45 clinical isolates was due to mutations in *MSH2*, a wild-type copy of *MSH2* was introduced into C23 and C45 at an ectopic location (Materials and Methods). Qualitative assessment of the mutation rate using the number of spontaneously arising 5-FOA colonies was used to assess complementation (Materials and Methods). The introduction of a wild-type copy of *MSH2* complemented the increased mutation rate phenotype of both the C23 and C45 isolates, suggesting that the mutations in *MSH2* identified by next-generation DNA sequencing were responsible for the enhanced mutation rate in these clinical isolates (Fig. 1B).

### Deletion of MMR components results in an elevated mutation rate.

In order to further investigate the contribution of single-base-pair mutations arising from errors in mismatch repair to microevolution, three strains with deletions in components of the mismatch repair pathway (the *msh2*Δ, *mlh1*Δ, and *pms1*Δ mutants) were obtained from the *Cryptococcus neoformans* H99 deletion collection (13). The levels of proliferation of the three mutants within lungs of mice had previously been shown to be increased in competition assays (13). The strains were backcrossed twice to the KN99**a** *MAT***a** congenic parent to remove unwanted melanin and mating differences known to exist in their background (13). Complemented strains were generated by reintroduction of the wild-type gene at an ectopic location using *Agrobacterium tumefaciens*-mediated transformation and resistance to G-418. Double mutants (*msh2*Δ *mlh1*Δ, *msh2*Δ *pms1*Δ, and *mlh1*Δ *pms1*Δ strains) were generated by crossing.

**FIG 2:**
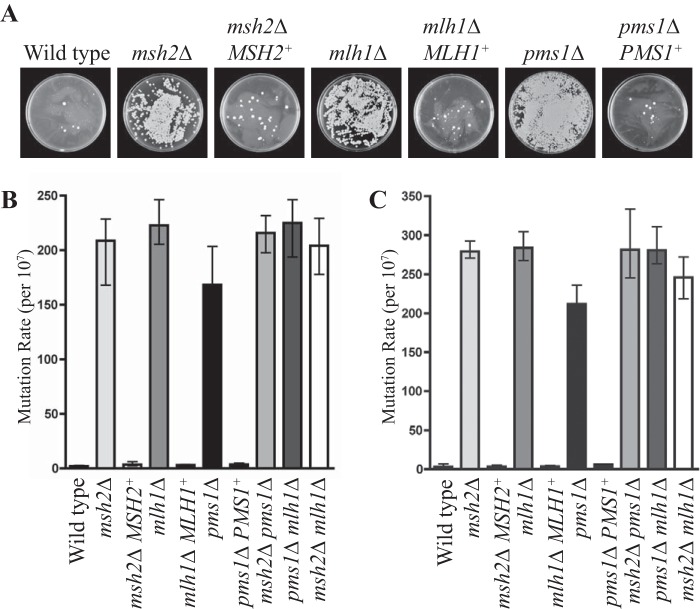
Deletion of three MMR components elevates mutation rates. (A) Spontaneous 5-FU-resistant colonies in the wild-type, *msh2*Δ, *msh2*Δ *MSH2*^*+*^, *mlh1*Δ, *mlh1*Δ *MLH1*^*+*^, *pms1*Δ, and *pms1*Δ *PMS1*^*+*^ strains. (B and C) Quantitative assessment of mutation rates using fluctuation analysis based on spontaneous resistance to 5-FOA (B) and 5-FU (C). The Lea-Coulson method of the median was used to estimate the number of mutations from the observed values of mutants across 20 independent parallel cultures.

Mutation rates were qualitatively assessed by observing the generation of spontaneously resistant 5-fluoroorotic acid (5-FOA) and 5-fluorouracil (5-FU) colonies in the mutant strains. 5-FU inhibits the growth of wild-type strains by inhibiting the activity of thymidylate synthetase, which affects pyrimidine synthesis and leads to an imbalance of intracellular dNTP pools. Compared to the wild-type and complemented strains, the *msh2*Δ, *mlh1*Δ, and *pms1*Δ mutants displayed an increase in levels of spontaneously resistant 5-FOA and 5-FU colonies suggestive of a higher mutation rate (Fig. 2A). The increased mutation rate was quantitatively assessed using fluctuation analysis, and the Lea-Coulson method of the median with FALCOR software was used to estimate the number of mutations from the observed values of mutants across the 20 independent parallel cultures on 5-FOA (Fig. 2B) and 5-FU (Fig. 2C) (24) (Materials and Methods). The *msh2*Δ, *mlh1*Δ, and *pms1*Δ mutants possessed mutation frequencies approximately 200-fold higher than those of the wild-type and complemented strains (Fig. 2B and C). Analysis of the double mutants indicated that there were no additive effects on mutation frequencies (Fig. 2B and C).

In addition to *MSH2*, the *C. neoformans* genome carries a further five MutS homologues (*MSH1*, *MSH3*, *MSH4*, *MSH5*, and *MSH6*). To test if the situation in *C. neoformans* is similar to that in *S. cerevisiae*, i.e., to test whether *MSH1*, *MSH4*, and *MSH5* do not have a role in nuclear DNA repair and whether *MSH3* and *MSH6* have redundant roles in mismatch repair, *MSH1*, *MSH3*, *MSH4*, and *MSH5* were individually deleted in KN99α by replacing the open reading frame via homologous recombination with a cassette conferring nourseothricin resistance (Materials and Methods). The mutation rate was assessed for each of the deletion strains using fluctuation analysis and resistance to 5-FOA (Materials and Methods). Deletion of *MSH1*, *MSH3*, *MSH4*, and *MSH5* did not affect the nuclear mutation frequency (see Fig. S1 in the supplemental material).

10.1128/mBio.00595-17.1 FIG S1  Deletion of *MSH1*, *MSH3*, *MSH4*, and *MSH5* does not affect nuclear mutation rates. Quantitative assessment of mutation rate using fluctuation analysis based on spontaneous resistance to 5-FOA. The Lea-Coulson method of the median was used to estimate the number of mutations from the observed values of mutants across 20 independent parallel cultures. Download FIG S1, EPS file, 1.2 MB. Copyright © 2017 Boyce et al. 2017 Boyce et al. This content is distributed under the terms of the Creative Commons Attribution 4.0 International license.

### MMR mutants have an increased proportion of transitions and mutations in homopolymeric tracts.

**FIG 3:**
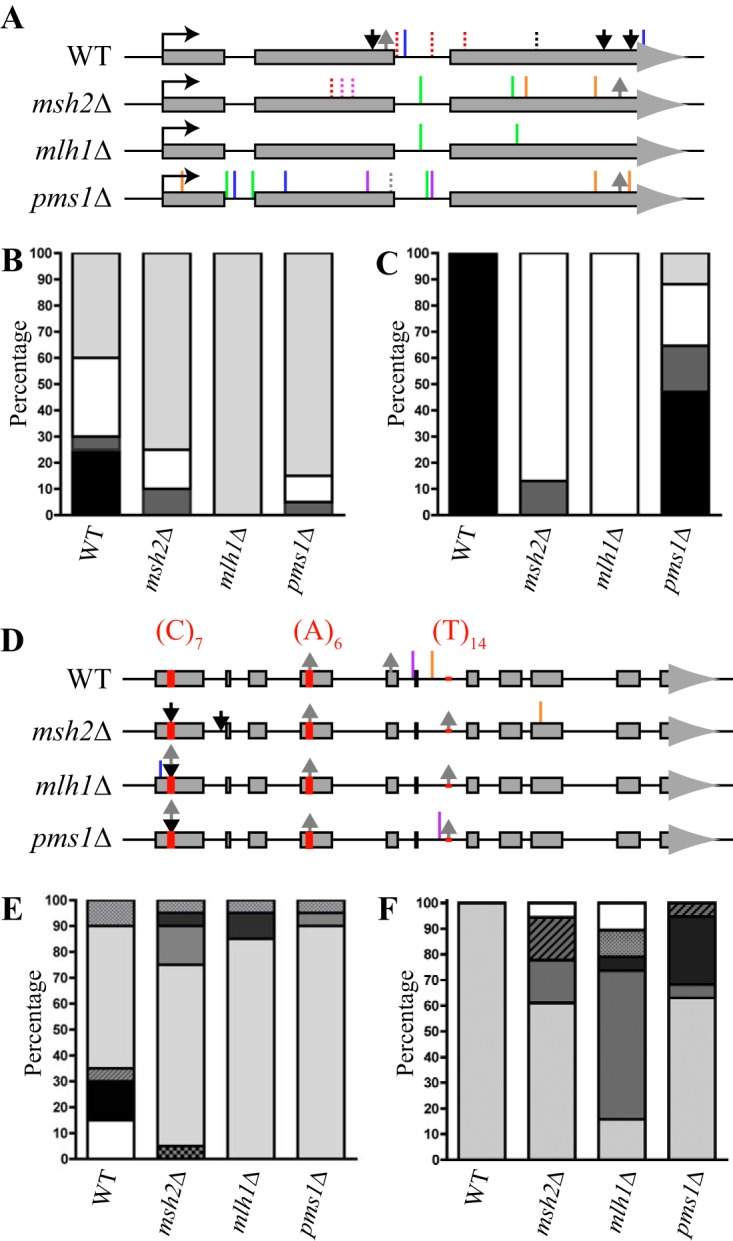
MMR mutants show an increased proportion of single-base-pair G-to-A transitions and an increased proportion of deletions in multiple types of homopolymeric tracts. (A) Schematic representation of the *URA5* gene indicating the locations and types of spontaneous mutations generated in the 5-FOA-resistant isolates derived from the wild-type (WT), *msh2*Δ, *mlh1*Δ, and *pms1*Δ strains. Insertions are indicated with black arrows, deletions with gray arrows, transitions as solid lines, and transversions as dashed lines. Mutations are as follows: red, G/T; black, G/C; pink, C/A; green, G/A; blue, T/C; orange, C/T; purple, A/G; gray, A/T. (B) Percentages of *URA5* insertions (black), deletions (dark gray), transversions (white), and transitions (light gray) in the WT, *msh2*Δ, *mlh1*Δ, and *pms1*Δ 5-FOA-resistant isolates. MMR mutants showed an increased proportion of single-base-pair transition mutations. (C) Percentage of transition types in *URA5* from the WT, *msh2*Δ, *mlh1*Δ, and *pms1*Δ 5-FOA-resistant isolates. The transition mutations are indicated as follows; black, T to C; dark gray, C to T; white, G to A; light gray, A to G. MMR mutants show an increased proportion of single-base-pair G/A transition mutations. (D) Schematic representation of the *FUR1* gene indicating the locations and types of spontaneous mutations generated in the WT, *msh2*Δ, *mlh1*Δ, and *pms1*Δ 5-FU-resistant isolates. Insertions are indicated with black arrows, deletions with gray arrows, and transitions as solid lines. Colors of transition mutations are as follows: G/A, blue; T/C, orange; C/T; purple, A/G. The three homopolymeric tracts in *FUR1* are indicated as red boxes. (E) Graph showing the percentages of *FUR1* with no mutations (white), large deletions (black), small deletions (hatched gray), small insertions (checkered gray), deletions in homopolymeric tracts (light gray), insertions in homopolymeric tracts (medium gray), and both deletions and insertions in homopolymeric tracts (dark gray) in the WT, *msh2*Δ, *mlh1*Δ, and *pms1*Δ 5-FU-resistant isolates. MMR mutants showed an increased proportion of deletions in homopolymeric tracts. (F) Graph of the percentages of *FUR1* deletions in the (A)_6_ homopolymer (light gray), deletions in the (A)_6_ homopolymers and (T)_14_ homopolymers (medium gray), deletions in the (C)_7_ homopolymer (dark gray), deletions in the (C)_7_ and (T)_14_ homopolymers (spotted gray), insertions in the (C)_7_ homopolymer (hatched gray), and insertions in the (C)_6_ homopolymer and deletions in the (T)_14_ homopolymer (white) in the WT, *msh2*Δ, *mlh1*Δ, and *pms1*Δ 5-FU-resistant isolates. MMR mutants showed an increased proportion of deletions in multiple types of homopolymeric tracts.

Mutations of the *URA5* and *FUR1* genes in *C. neoformans* are the most common cause of resistance to 5-FOA and 5-FU, respectively (12, 26). In order to determine the types of mutations generated in the absence of mismatch repair, *URA5* and *FUR1* were sequenced from 20 5-FOA-resistant and 20 5-FU-resistant strains isolated from the wild-type and *msh2*Δ, *mlh1*Δ, and *pms1*Δ mutant strains derived from the independent parallel cultures used in the fluctuation analysis. *URA5* from wild-type 5-FOA-resistant strains contained a variety of mutations, including small insertions or deletions, transversions, and transitions (Fig. 3A). The *msh2*Δ, *mlh1*Δ, and *pms1*Δ mutants displayed a shift in the mutation profile of *URA5* (Fig. 3A and B). In comparison to the wild-type results, *URA5* from *msh2*Δ, *mlh1*Δ, and *pms1*Δ 5-FOA-resistant strains showed a reduction in the number of insertion, deletion, and transversion mutations and an increase in the number of transition mutations (from 40% in the wild type to 75%, 100%, and 85% in the mutants, respectively) (Fig. 3B). In addition, the types of transition mutations generated in the *msh2*Δ, *mlh1*Δ, and *pms1*Δ strains differed from the wild-type results. In the wild-type strain, 100% of the transition mutations generated were T-to-C transitions. In contrast, the *msh2*Δ and *mlh1*Δ mutants generated predominately G-to-A transitions (Fig. 3C). This altered the positions of mutations to both coding and noncoding regions, as G-to-A transitions frequently occurred at intron splice sites and sites of lariat formation (Fig. 3A). In contrast to the wild-type strain and the *msh2*Δ and *mlh1*Δ mutants, the *pms1*Δ mutant produced all types of transitions (Fig. 3C).

Unlike *URA5*, *FUR1* contains a number of homopolymeric tracts: (C)_7_ at +81 to +87, (A)_6_ at +460 to +465, and (T)_14_ at +941 to +954 (Fig. 3D). The *FUR1* gene sequenced from 5-FU-resistant strains derived from the wild-type strain contained a variety of mutations, including large and small deletions, transitions, and deletions in homopolymeric tracts (Fig. 3D). Similarly to the observation for *URA5*, the *msh2*Δ, *mlh1*Δ, and *pms1*Δ mutants displayed a shift in the mutation profile of *FUR1* compared to that of *FUR1* of the wild type (Fig. 3E). In comparison to the wild-type results, *FUR1* from *msh2*Δ, *mlh1*Δ, and *pms1*Δ 5-FU-resistant strains possessed a reduction in the numbers of large deletions and an increase in the numbers of deletions and insertions in homopolymeric tracts (from 55% in the wild-type strain to 90%, 95%, and 95% in the mutants, respectively) (Fig. 3E). The numbers of transition mutations in *FUR1* between the strains remained equivalent, suggesting that mutations in homopolymeric tracts occur more frequently than transitions. In addition, the type of mutations generated in homopolymeric tracts in the *msh2*Δ, *mlh1*Δ, and *pms1*Δ strains differed from that seen with the wild type. In the wild type, 100% of the mutations generated in homopolymeric tracts were deletions in the (A)_6_ homopolymer. In contrast, the *msh2*Δ, *mlh1*Δ, and *pms1*Δ mutants possessed a variety of types of mutations in homopolymeric tracts in addition to deletions in the (A)_6_ homopolymer, including deletions in the (C)_7_ and (T)_14_ homopolymers and an insertion in the (C)_7_ homopolymer (Fig. 3F). Also, unlike the wild type, in which 100% of the mutations in homopolymeric tracts involved only one tract, *FUR1* from the 5-FU-resistant *msh2*Δ, *mlh1*Δ, and *pms1*Δ strains possessed mutations in multiple homopolymeric tracts (22%, 79%, and 5%, respectively) (Fig. 3F).

### Deletion of MMR components results in only minor sensitivity to DNA-damaging agents and oxidative stress.

**FIG 4:**
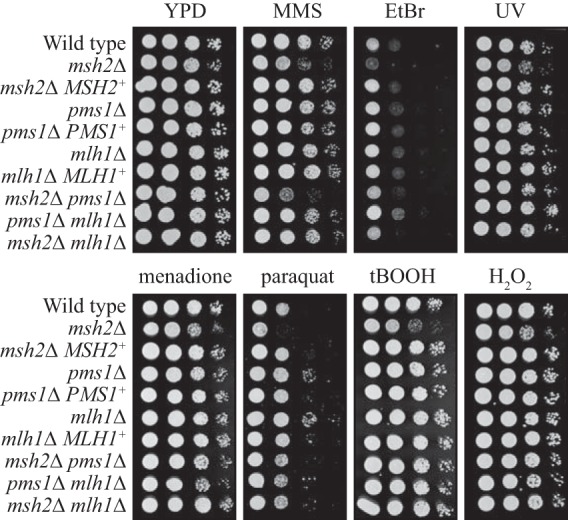
Deletion of MMR components causes only minor sensitivity to DNA-damaging agents and oxidative stress. Strains were cultured overnight in YPD medium, 10-fold serially diluted, and plated onto YPD medium with or without the following stress agents: 0.04% methyl methanesulfonate (MMS), 0.06% ethidium bromide solution (EtBr), 0.25 mM menadione, 0.25 mM paraquat dichloride hydrate (paraquat), 0.4 mM tert-butyl hydroperoxide solution (tBOOH), 5 mM hydrogen peroxide solution (H_2_O_2_). One set of plates was exposed to UV light (120 J m^−2^). Plates were incubated at 28°C for 2 days.

To explore further the role of MMR components in DNA repair, the *msh2*Δ, *mlh1*Δ, and *pms1*Δ mutants were assessed for phenotypes associated with sensitivity to or tolerance of DNA-damaging agents. Strains were cultured on a selection of DNA-damaging chemicals, including those associated with oxidative stress, or were treated with UV light (UV) (Fig. 4). The *msh2*Δ, *msh2*Δ *pms1*Δ, and *msh2*Δ *mlh1*Δ mutants showed a minor sensitivity to the following DNA-damaging agents (which affected DNA replication): methyl methanesulfonate (MMS), ethidium bromide (EtBr), and UV (Fig. 4). MMS methylates DNA to stall replication forks, EtBr affects replication by deforming the DNA by intercalation, and UV causes pyrimidine dimers to prevent DNA replication. The *msh2*Δ mutant also showed minor sensitivity to the following chemicals (which generate reactive oxygen species [ROS]): menadione, paraquat dichloride hydrate (paraquat), tert-butyl hydroperoxide (tBOOH), and hydrogen peroxide (H_2_O_2_) (although this was not observed in any of the double mutants) (Fig. 4). In contrast, the *pms1*Δ and *mlh1*Δ mutants showed increased tolerance of paraquat (Fig. 4). ROS predominately introduces base or sugar damage leading to single-stranded break formation. Minor or no phenotypes are expected on ROS as oxidative damage is mainly repaired by the base excision repair (BER) pathway, although MMR has been shown to play a role in repairing oxidation of guanine to 8-oxo-7,8-dihydroguanine (8-oxoG) (27).

### Disruption of the mismatch repair pathway leads to rapid microevolution.

**FIG 5:**
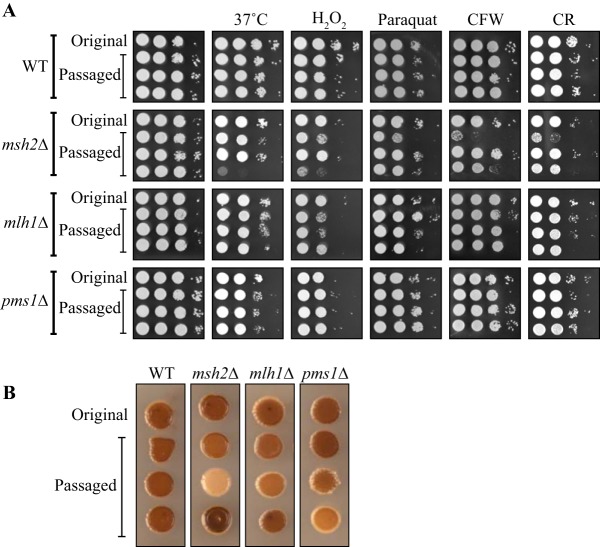
Disruption of the mismatch repair pathway leads to rapid microevolution of new phenotypes. Comparisons of the original wild-type strain and the *msh2*Δ, *mlh1*Δ, and *pms1*Δ mutants with 3 strains derived from independent passaging for approximately 600 generations with population bottlenecks every 90 generations were performed. (A) Strains grown at 37°C or at 28°C for 2 days on 5 mM hydrogen peroxide solution (H_2_O_2_), 0.25 mM paraquat dichloride hydrate (paraquat), 1.5 mg/ml calcofluor white (CFW), and 5 mg/ml Congo red (CR). (B) Melanization on l-DOPA medium. These experiments were repeated multiple times with consistent results.

In order to investigate whether the increased mutation rate observed in the MMR mutants results in an increased rate of microevolution and phenotypic change, the wild-type strain and the *msh2*Δ, *mlh1*Δ, and *pms1*Δ mutants were passaged 3 times independently in culture for approximately 600 generations with population bottlenecks every 90 generations (Materials and Methods). Passaging through population bottlenecks allows nonlethal mutations to accumulate as if they were neutral by mitigating the effect of selection. The resultant strains were compared to the original nonpassaged mutants and assessed for phenotypes that are associated with the ability to grow *in vivo*, including growth at 37°C, resistance to oxidative stress (paraquat and H_2_O_2_), alterations in the cell wall (Congo red and calcofluor white stress), and melanization. No phenotypic differences were observed between the original wild-type strain and the 3 passaged wild-type strains at 37°C or on H_2_O_2_, paraquat, calcofluor white, or Congo red (Fig. 5A). In contrast, the phenotypes of the passaged *msh2*Δ and *mlh1*Δ mutants differed dramatically under these conditions from the original mutants and from each other, suggesting that the rate of microevolution was occurring more rapidly in these strains than in wild type (Fig. 5A). The *msh2*Δ strain showed more phenotypic variability than the *mlh1*Δ strain (Fig. 5A). The passaged *pms1*Δ strains were phenotypically the same as the original mutant and the wild type (Fig. 5A). Likewise, no differences between the original and passaged wild-type strains in melanization were observed (Fig. 5B). In contrast, the melanization results differed dramatically in the passaged *msh2*Δ strains compared to the original mutant, with each strain exhibiting a decrease in melanization, an almost complete absence of melanization, and an increase in melanization (Fig. 5B). The passaged *mlh1*Δ and *pms1*Δ strains showed differences in melanization compared to the original mutants also, though the differences were not as extreme as those seen with the *msh2*Δ strains (Fig. 5B).

### Deletion of MMR components leads to rapid resistance to antifungal agents.

**FIG 6:**
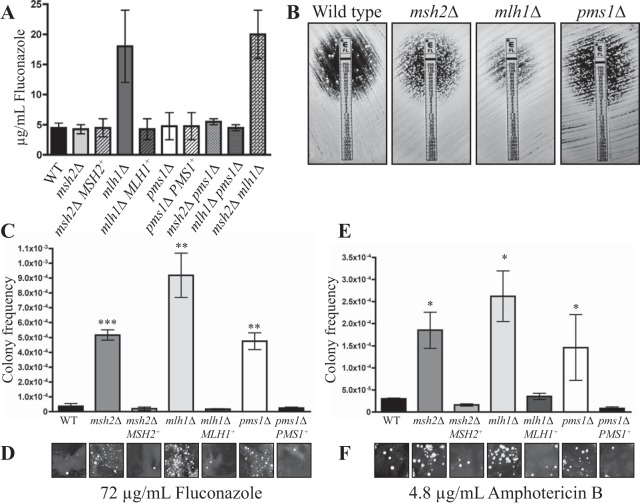
Deletion of MMR components leads to rapid resistance to antifungal agents. (A) Fluconazole MICs determined by Etests. (B) Fluconazole Etests of the wild-type strain and the *msh2*Δ, *mlh1*Δ, and *pms1*Δ mutants showing spontaneously arising fluconazole-resistant colonies in the zone of clearing. (C) Quantification of the number of spontaneously resistant colonies arising on 72 µg/ml fluconazole (16× MIC) media. Asterisks indicate statistical significance determined using a two-tailed Student’s *t* test (***, *P* < 0.0005; **, *P* < 0.005). (D) Spontaneously resistant colonies arising on 72 µg/ml fluconazole differed in size. (E) Quantification of the number of spontaneously resistant colonies arising on 4.8 µg/ml amphotericin B (32× MIC). Asterisks indicate statistical significance determined using a two-tailed Student’s *t* test (*, *P* < 0.05). (F) Spontaneously resistant colonies arising on 4.8 µg/ml amphotericin B differed in size.

To investigate the clinical relevance of disrupting mismatch repair in *C. neoformans* and the consequences of rapid microevolution, the fluconazole MIC was determined for the mutant strains using Etests (Materials and Methods). The *msh2*Δ and *pms1*Δ mutants had a MIC equivalent to that seen with the wild-type and complemented strains (Fig. 6A). The *mlh1*Δ mutant possessed much higher resistance to fluconazole than the wild-type, *msh2*Δ and *pms1*Δ mutant, and *mlh1*Δ *MLH1*^+^ complemented strains (Fig. 6A). This increase in fluconazole resistance was also observed in the *msh2*Δ *mlh1*Δ double mutant but not in the *pms1*Δ *mlh1*Δ mutant (Fig. 6A). All of the MMR mutants showed an increase in the number of spontaneously arising fluconazole-resistant colonies in the zone of clearing (Fig. 6B).

To quantify the increase in the number of spontaneously arising fluconazole-resistant colonies, the colony frequency on 72 µg/ml fluconazole (16× MIC) was calculated as described in reference 21. The *msh2*Δ, *mlh1*Δ, and *pms1*Δ mutants all showed a statistically significant increase in the frequency of fluconazole-resistant colonies (Fig. 6C). The *mlh1*Δ mutant displayed a higher number of spontaneously arising fluconazole-resistant colonies than the *msh2*Δ and *pms1*Δ mutants that was probably a reflection of the higher MIC (Fig. 6C and D). Unlike the wild-type results, colonies of the *msh2*Δ, *mlh1*Δ, and *pms1*Δ mutants differed dramatically in size, suggesting that newly acquired fluconazole resistance was occurring frequently throughout the time period of the experiment (Fig. 6D).

The number of colonies arising that were spontaneously resistant to an antifungal unrelated to the azoles, amphotericin B, was also assessed. The colony frequency was calculated on 4.8 µg/ml amphotericin B (32× MIC) using the previously determined MIC for KN99α (21, 28). Similarly to the rapid emergence of resistance to fluconazole, the *msh2*Δ, *mlh1*Δ, and *pms1*Δ mutants all showed a statistically significant increase in the frequency of amphotericin B-resistant colonies compared to the wild type, and the mutant colonies also differed in size from those seen with the wild-type and complemented strains (Fig. 6E and F). The *mlh1*Δ mutant also displayed a higher number of spontaneously arising amphotericin B-resistant colonies than the *msh2*Δ and *pms1*Δ mutants (Fig. 6E).

To assess if the single mutations were resulting in multidrug-resistant colonies, 20 fluconazole-resistant and 20 amphotericin B-resistant colonies isolated from the wild-type strain or the *msh2*Δ mutant were grown on 72 µg/ml fluconazole and 4.8 µg/ml amphotericin B. Fluconazole-resistant strains derived from either wild-type or *msh2*Δ strains were not resistant to amphotericin B and vice versa (data not shown).

**FIG 7:**
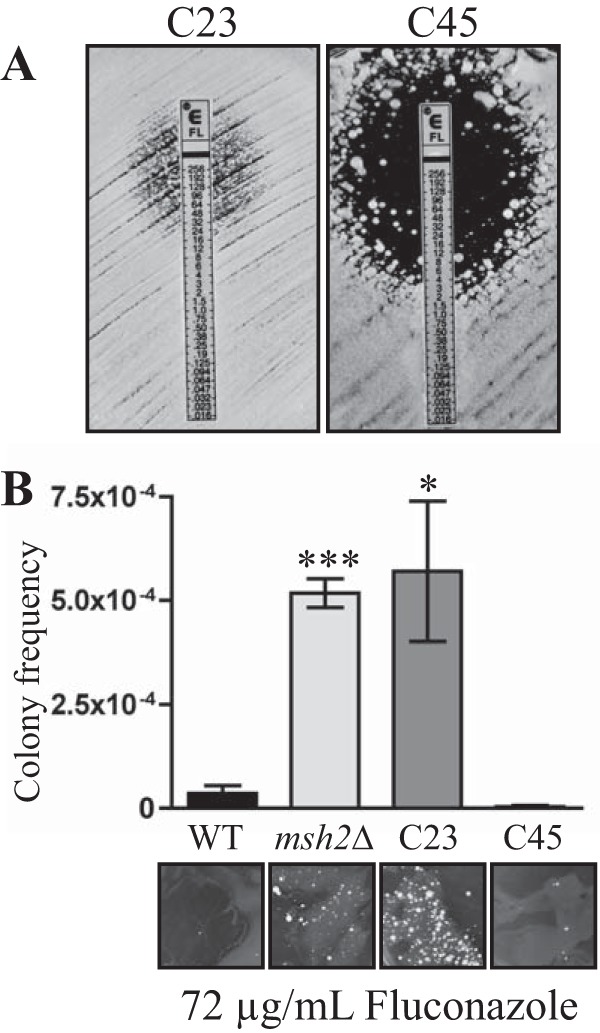
Two clinical mismatch repair mutants display increased spontaneous resistance to antifungal agents. (A) Fluconazole Etests of the C23 and C45 clinical isolates, showing MIC and spontaneously arising fluconazole-resistant colonies in the zone of clearing. (B) Quantification of the number of spontaneously resistant colonies arising on 72 µg/ml fluconazole (16× MIC). Asterisks indicate statistical significance determined using a two-tailed Student’s *t* test (***, *P* < 0.0005; *, *P* < 0.05). Lower panels illustrate colony formation on fluconazole.

To determine if the increased mutation rate had any bearing on clinical treatments, the fluconazole MIC was also determined for the C23 and C45 clinical isolates strains using Etests. The C23 isolate possessed a much higher resistance to fluconazole (32 ± 0.0 µg/ml) than the wild-type strain (4.5 ± 0.8 µg/ml) (Fig. 6B) and also displayed an increase in the number of spontaneously arising fluconazole-resistant colonies in the zone of clearing (Fig. 7A). In contrast, the C45 isolate possessed a MIC lower than that of the wild-type strain (3.0 ± 1.0 µg/ml) and very few spontaneously arising fluconazole-resistant colonies in the zone of clearance (Fig. 7A). To quantify the increase in the number of spontaneously arising fluconazole-resistant colonies, the colony frequency was calculated on 72 µg/ml fluconazole (16× MIC) as described in reference 21. The C23 isolate showed a statistically significant increase in the frequency of spontaneously arising fluconazole-resistant colonies (Fig. 7B).

### Deletion of *PMS1*, but not *MSH2* or *MLH1*, results in a reduction in virulence.

**FIG 8:**
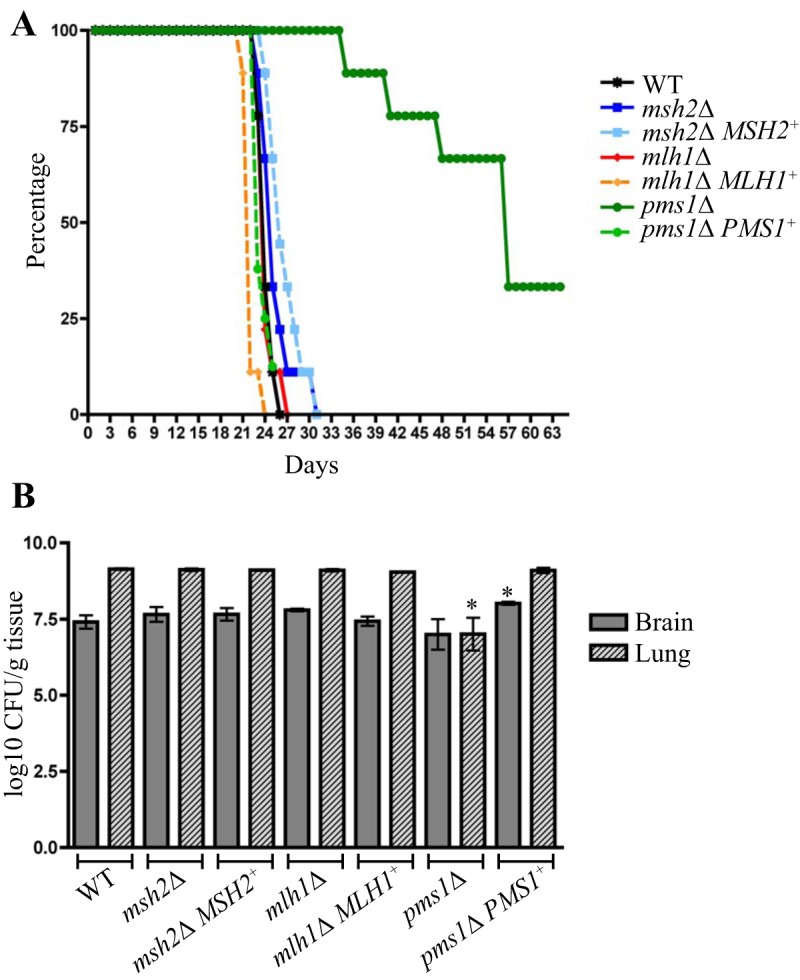
Deletion of *PMS1*, but not *MSH2* or *MHL1*, results in a reduction in *C. neoformans* virulence. (A) Percent survival of wild-type (WT), MMR mutant, and complementation strains in a murine inhalation virulence assay. (B) CFU levels measured from brain and lung tissue of mice at sacrifice. Asterisks indicate statistical significance determined using a two-tailed Student’s *t* test (*, *P* < 0.05).

In a large-scale analysis of virulence, 1,201 defined deletion mutants were bar-coded with 1 of 48 unique signature tag DNA sequences, pooled in groups of 48, and inoculated into the lungs of mice, thus allowing many mutants to be tested in a single-animal infection (13). The *msh2*Δ, *mlh1*Δ, and *pms1*Δ mutants showed increased proliferation in this lung assay of cryptococcosis (13). However, as increased proliferation could be attributed to increased adaptation to the host, to a selective advantage in a competitive environment, or to a population bottleneck, virulence of the MMR mutants was also assessed in a conventional murine inhalation virulence assay. In this assay, the virulence, or fungal load in the lung or brain, of the *msh2*Δ and *mlh1*Δ mutants did not significantly differ from that seen with the wild-type or complemented strains (Fig. 8). In contrast, the *pms1*Δ mutant showed a reduction in virulence and fungal load in the lung, compared to the wild type and the *pms1*Δ *PMS1*^+^ complemented strain (Fig. 8).

## DISCUSSION

Transmission of an organism’s genetic material through mitosis is extremely accurate due to the activity of a number of systems that detect the introduction of incorrect nucleotides during DNA replication. However, mutations that arise through errors in replication from environmental stresses are biologically important since they provide variations upon which selection can act. Those which confer fitness defects are removed by natural selection, whereas others provide an opportunity for adaptive evolution to occur. One mechanism regulating the control of mutagenesis is the mismatch repair pathway. Mutation of genes encoding components of this pathway can provide an increased mutation rate to enhance the possibility of generating phenotypic traits that could increase survival of a microbial lineage.

This study has shown that mutations in MMR components in the human fungal pathogen *C. neoformans* result in an increase of over 200-fold in the mutation rate compared to the wild-type rate. This corresponds to approximately 1 mutation per genome per generation in contrast to 1 mutation every few hundred generations for the wild type. The *msh2*Δ mutation rate is equivalent to that observed in *msh2*Δ mutants in *S. cerevisiae* (24). The indistinguishable mutation rates of the *msh2*Δ, *mlh1*Δ, and *pms1*Δ *C. neoformans* mutants, individually or as double gene deletion strains, indicates that, as in *S. cerevisiae*, the majority of mismatches repaired by MutSα/β require a Mlh1-Pms1 MutLα heterodimer. The *C. neoformans* MMR mutants also displayed a shift in the mutational profile compared to the wild type. The mutants showed an excess of transition mutations, specifically, G-to-A transitions in the *msh2*Δ and *mlh1*Δ mutants, and an increase in single-nucleotide deletions and insertions at homopolymeric tracts. The higher mutation rates compared to those seen with 5-FOA-resistant strains determined by the isolation of 5-FU-resistant strains likely reflects the fact that mutations at homopolymeric tracts represent the primary mutational event in MMR mutants. This hypothesis is also supported by the decreased transition rate observed in the *fur1* mutants compared to the *ura5* mutants, where they were the predominant mutation. A similar shift in mutational profile is also observed in *S. cerevisiae msh2*Δ mutants, in which an equivalent shift to transitions is observed and a large number of single nucleotide variations (−1 to +1) occur within homopolymeric tracts (24, 25). In *S. cerevisiae*, single nucleotide variations are also detected within dinucleotide/trinucleotide tracts (microsatellites) (24, 25). These types of mutations could not be detected with the assays used in this *C. neoformans* study. This is one of the limitations of using genetic reporter assays to measure mutation rates and to analyze the mutational spectrum. Rates of phenotypically silent mutations are not measured, as evidenced by the frequent deletion of nucleotides in the (T)_14_ homopolymer of *FUR1* in the 5-FU-resistant *msh2*Δ, *mlh1*Δ, and *pms1*Δ isolates, in combination with mutations in the (C)_7_ and (A)_6_ homopolymers, as the (T)_14_ homopolymer falls within an intron and is therefore silent (Fig. 3D). Despite the increased mutation rate and the shift in the mutational profile, the MMR mutants did not show a decrease in viability *in vitro* or for the *msh2*Δ and *mlh1*Δ mutants in a mouse model of disease. The majority of mutations are likely to occur in noncoding DNA or to be silent, and it is expected that only a minute fraction would be deleterious. Overall, these results suggest that the role of Msh2, Mlh1, and Pms1 in nuclear mismatch repair is conserved in *C. neoformans*.

The types of mutations generated suggest that Msh2, Mlh1, and Pms1 correct errors in nuclear DNA arising both from errors in replication and from oxidative damage. G-to-A transitions, followed by G-to-T transversions, are the most commonly observed mutations arising from oxidative damage to DNA but can also be generated through misincorporation of dUTP instead of dTTP during replication or deamination of cytosine (29). The resulting uracil in DNA is typically removed by the homologue of the Ung1 uracil DNA N-glycosylase of the base excision repair (BER) pathway (30). G-to-A transitions also occur by the deamination of methylated cytosine to thymine. However, none of the G-to-A transitions observed in the MMR mutants were located at CpG sites, the typical sites of cytosine methylation, suggesting that the increase in this transition type is not due to a failure to repair damaged methylated cytosine. A lack of repair of guanine oxidized to 8-oxo-7,8-dihydroguanine (8-oxoG) can also result in an increased number of G-to-A transitions (depending on the sequence context) but normally results in an abundance of G-to-T transversions, which was not observed in the MMR mutants (27, 29). Therefore, it is not clear why there was an abundance of G-to-A transitions in the MMR mutants. DNA slippage events and misincorporation of nucleotides during the synthesis of the leading and lagging strands by the DNA polymerases ε (Pol2) and ∂ (Pol3), respectively, give rise to single nucleotide variations within homopolymeric tracts and transition and transversion mutations (31, 32). However, the majority of mutations are generated by errors during lagging-strand synthesis by DNA polymerase ∂ (Pol3), which shows inefficient proofreading and, consequently, lower fidelity than that seen with DNA polymerase ε (Pol2) (31). MMR is more efficient at repairing lagging-strand errors generated by DNA polymerase ∂ (Pol3) as the MutSα/β complex is directed to the 5′ ends of Okazaki fragments and is tethered to the proliferating cell nuclear antigen (PCNA), a DNA clamp that acts as a processivity factor for DNA polymerase ∂ (Pol3) (31–33).

It is interesting that the mutational profile and phenotype of the *pms1*Δ mutant differ from those of the *msh2*Δ and *mlh1*Δ mutants, and this may relate to the reason that the *pms1*Δ mutant strain shows attenuation of virulence whereas the other two mutants do not. Although the *pms1*Δ mutant displays a shift in the mutational profile to more transition mutations and mutations in homopolymeric tracts, like the *msh2*Δ and *mlh1*Δ mutants, the types of transition mutations generated differ. This suggests that Pms1 is playing a role in addition to that of the MutLα heterodimer in nuclear mismatch repair, possibly through interaction with the PCNA. The range of transitions generated in the *pms1*Δ mutant may result in more deleterious mutations than in *msh2*Δ and *mlh1*Δ mutants, as a greater range of codons can be mutated and as C-to-T transitions also result in premature stops, unlike G-to-A transitions. In addition, little phenotypic variation was observed in the *pms1*Δ strains passaged for 600 generations, suggesting that most of the mutations that occurred were lethal and were removed from the population by natural selection. Thus, the reduction in virulence observed in the *pms1*Δ mutant likely arises due to decreasing survival over time. In contrast, passaging the *msh2*Δ and *mlh1*Δ mutants through population bottlenecks allowed nonlethal mutations to accumulate as if they were neutral by mitigating the effect of selection.

Mutations resulted in measurable phenotypic change, suggesting that those arising in the *msh2*Δ and *mlh1*Δ mutants provide a powerful mode of rapid microevolution in *C. neoformans*. We observed no difference in proliferation or virulence in the *msh2*Δ and *mlh1*Δ mutants in a standard murine inhalation virulence assay. This is in contrast to the original analysis of these mutants, which showed they had increased proliferation *in vivo* (13). In that experiment, however, 48 pooled mutants were simultaneously inoculated into mice to allow large-scale screening and a less virulent genetic background was used. It is possible that an increase in proliferation would be observed only in a strain with lower virulence than that seen in the KN99 background, when the mutants are in a competitive environment, or when a small number of cells are inoculated, effectively creating a population bottleneck. Similarly to *C. neoformans*, equivalent levels of mice colonization and infection were also observed between the wild type and the *msh2*Δ mutant in a standard *C. glabrata* virulence assay (21). Rather than having a direct impact on the virulence composition of isolates, MMR mutations are more likely to be a significant factor in allowing outbreaks to occur via the generation of hypervirulent strains from less-virulent ancestors and by allowing recurrence of cryptococcal meningitis through the generation of antifungal resistance.

Microevolution by single-base-pair mutations may potentially play a pivotal role in the development of infectious outbreaks from less-virulent ancestors. One example comes from the outbreak of cryptococcosis that began in the Pacific Northwest of the United States and western Canada in the late 1990s. One of the three clonal subpopulations causing the outbreak consisted of two distinct groups: a group of less-virulent strains containing a frameshift mutation in *MSH2* and a group of highly virulent outbreak strains in which the *MSH2* mutation is hypothesized to have reverted (34). This suggests that a transient mutator phenotype could have contributed to microevolution and adaptation of virulence in this cryptococcosis outbreak. A transient mutator provides a scenario by which mutators can benefit temporarily from elevated mutation frequencies for adaptation and yet can reduce the risk of accumulating deleterious mutations.

The mutator phenotype of the *C. neoformans* MMR mutants also promoted rapid development of antifungal drug resistance, as observed by the frequently arising fluconazole- and amphotericin B-resistant colonies. Patients treated for cryptococcal meningitis often relapse due to gain of resistance to antifungal drugs (35). Mutations in *MSH2* provide the pathogen with an opportunity for the rapid evolution of resistance to antifungal drugs and could be a cause of patient relapse. Recently, in a study investigating the causes of recurrence of meningitis due to *C. neoformans*, one isolate with a mutation in the *MSH2* gene was identified (35). Deletions of *MSH2* in *C. glabrata* also result in high levels of spontaneous antifungal resistance *in vitro*, in addition to an increase in the occurrence of resistance to caspofungin arising during *in vivo* growth (21). *MSH2* mutations are present in a higher percentage in fluconazole-resistant clinical isolates than in susceptible strains in *C. glabrata* (21). In addition, a comparison of paired or triplet isolates from the same patient showed that the *MSH2* mutation predated the emergence of fluconazole resistance (21). Likewise, deletion of *MSH2* and *PMS1* in *C. albicans* also resulted in resistant colonies arising in the inhibition ellipse of fluconazole Etests, although the rate was not as high as that observed in *C. neoformans* or *C. glabrata* (as expected due to the diploid state of *C. albicans*) (36). Antifungal resistance in *C. neoformans* is often associated with changes in karyotype (reviewed in reference 6). Fluconazole heteroresistance occurs in *C. neoformans* via successive duplication of chromosomes 1, 4, 10, and 14 on increasing concentrations of fluconazole. These disomic chromosomes allow the rapid emergence of fluconazole resistance; however, the disomic chromosomes are lost when fluconazole is removed, presumably due to reduced fitness as evidenced by reduced proliferation and virulence (10). The emergence of antifungal resistance via single-base-pair mutations in MMR mutants may provide *C. neoformans* with another option that results in stable, long-term fluconazole resistance without associated fitness defects.

This study showed that a number of *C. neoformans* clinical isolates carry mutations in MMR components that give rise to a hypermutation phenotype, high levels of fluconazole resistance, and the ability to rapidly generate spontaneous fluconazole-resistant subisolates. In other microbial populations, retaining a proportion of mutator cells in the population is thought to provide a selective advantage *in vivo*. Fifty-five percent of *C. glabrata* clinical isolates recovered from patients are *msh2* mutants (other components of the MMR pathway were not sequenced) (21). In addition, the bacterial pathogens *Staphylococcus aureus*, *Eschericha coli*, and *Salmonella enterica* retain a larger-than-expected proportion of mutator cells in their populations with defects in components of MMR, and 20% of *Pseudomonas aeruginosa* isolates from cystic fibrosis patients are mutators compared to 0% of environmental isolates (17–20). This suggests that this adaptive mechanism may be conserved in a broad variety of pathogens. Whether *C. neoformans* clinical populations contain a high proportion of MMR mutants has yet to be determined and requires investigation of larger sample sizes for valid analysis of the proportion of clinical versus environmental isolates that carry an MMR mutation. What is clear is that mutations in MMR components provide *C. neoformans* with an additional mechanism enabling microevolution to occur and that this has implications for the development of antifungal resistance.

## MATERIALS AND METHODS

### Plasmid construction.

*MSH2*, *PMS1*, and *MLH1* were amplified from genomic DNA isolated from strain KN99α with primers ALID2273 and ALID2274 (*MSH2*), KB001 and KB002 (*MLH1*), and ALID2277 and ALID2278 (*PMS1*) (primer sequences are presented in Table S1 in the supplemental material) and were cloned into TOPO pCR2.1 (Invitrogen, Life Technologies, Inc., Grand Island, NY) to create plasmids KBG016, KBG006, and KBG017. Complementation constructs for *MSH2* (KBG013), *MLH1* (KBG018), and *PMS1* (KBG014) were generated by cloning the BamHI, SpeI, and SpeI/XbaI fragments from KBG016, KBG006, and KBG017, respectively, into *Agrobacterium* transformation vector pPZP-NEO11 (KBG012) digested with either BamHI or XbaI.

10.1128/mBio.00595-17.2 TABLE S1  Primers used in this study. Download TABLE S1, DOC file, 0.04 MB. Copyright © 2017 Boyce et al. 2017 Boyce et al. This content is distributed under the terms of the Creative Commons Attribution 4.0 International license.

*MSH1*, *MSH3*, *MSH4*, and *MSH5* were amplified from KN99α DNA using primers KB012 and KB013 (*MSH1*), KB020 and KB021 (*MSH3*), KB028 and KB029 (*MSH4*), and KB036 and KB037 (*MSH5*) (Table S1) and were cloned into pCR2.1 TOPO to create plasmids KBG001, KBG002, KBG003, and KBG004. Deletion constructs KBG028 (*msh1*Δ::*NAT*), KBG029 (*msh3*Δ::*NAT*), KBG030 (*msh4*Δ::*NAT*), and KBG031 (*msh5*Δ::*NAT*) were generated by PCR using TOPO pCR2.1 plasmids and primers KB150 and KB151 (*MSH1*), KB133 and KB134 (*MSH3*), KB152 and KB153 (*MSH4*), and KB154 and KB155 (*MSH5*) and by ligation to the nourseothricin acetyltransferase (NAT) resistance cassette generated with 5′ phosphorylated primers KB146 and KB147 (Table S1).

### Strains and growth conditions.

Strains used in this study are listed in Table S2. *C. neoformans* strain KN99α was used as the representative wild-type strain. Clinical isolates C23 and C45 were obtained from reference 22. The *msh2*Δ, *mlh1*Δ, and *pms1*Δ strains were obtained from a collection of deletion strains (13). These three strains were backcrossed twice to a *MAT***a** congenic parent to remove unwanted melanin and mating differences known to exist in their background (13, 37) to create strains AISVCN195, AISVCN196, and AISVCN198. Double mutants (mutants AISVCN206 *msh2*Δ *mlh1*Δ, AISVCN202 *msh2*Δ *pms1*Δ, and AISVCN204 *mlh1*Δ *pms1*Δ) were generated by a crossing strategy. The *msh2*Δ *MSH2*^*+*^ (KBCN008), *mlh1*Δ *MLH1*^*+*^ (KBCN0013), and *pms1*Δ *PMS1*^*+*^ (KBCN0021) complemented strains were generated by *Agrobacterium*-mediated transformation of *C. neoformans* strains AISVCN195 (*msh2*Δ), AISVCN196 (*mlh1*Δ), and AISVCN198 (*pms1*Δ) with plasmids KBG013, KBG018, and KBG014 using a method previously described (38). Transformants were selected on yeast extract-peptone-dextrose (YPD) medium containing cefotaxime (Sigma-Aldrich, Castle Hill, Australia) (200 µg/ml) and geneticin (G418) (Gibco Life Technologies, Inc., Scoresby, Australia) (50 µg/ml). Strains KBCN0029 (*msh1*Δ), KBCN0031 (*msh3*Δ), KBCN0045 (*msh4*Δ), and KBCN0035 (*msh5*Δ) were generated by precipitating the PCR-amplified deletion cassettes from KBG028 (amplified with KB012 and KB013), KBG029 (amplified with KB020 and KB021), KBG030 (amplified with KB028 and KB029), and KBG031 (amplified with KB036 and KB037) onto gold beads and transforming them into strain KN99α using a PDS/He-1000 biolistic apparatus (Bio-Rad, Hercules, CA [39]) and were plated onto YPD medium with nourseothricin (Jena Bioscience, Germany) (100 µg/ml). All gene replacements were confirmed by PCR.

10.1128/mBio.00595-17.3 TABLE S2  Strains used in this study. Download TABLE S2, DOC file, 0.1 MB. Copyright © 2017 Boyce et al. 2017 Boyce et al. This content is distributed under the terms of the Creative Commons Attribution 4.0 International license.

Isolates C23 and C45 were transformed with plasmids KBG013, KBG018, and KBG014 by *Agrobacterium*-mediated transformation to test complementation with wild-type copies of *MSH2*, *MLH1*, and *PMS1*, respectively. Transformants were selected on YPD medium containing cefotaxime (200 µg/ml) and geneticin (G418) (50 µg/ml).

*C. neoformans* strains were routinely cultured in yeast extract-peptone-dextrose (YPD) medium with or without 2% agar medium and stored as glycerol stocks at −80°C.

### Mutation rate analysis.

Spontaneous resistance to 5-fluoroorotic acid (5-FOA) and 5-fluorouracil (5-FU) was used to measure mutation frequency by fluctuation analysis. For each strain, 1 × 10^5^ cells from an overnight YPD culture were used to inoculate 20 separate YPD cultures. After growth for 48 h in a roller drum at room temperature, 1 × 10^7^ cells were plated onto yeast nitrogen base (YNB)–0.05 mg/ml uracil–1 mg/ml 5-FOA or onto YPD medium–0.25 mg/ml 5-FU. The numbers of spontaneously arising colonies were counted from the 20 independent cultures, and the Lea-Coulson method of the median was used with FALCOR software to estimate the number of mutations from the observed values of mutants across the parallel cultures (23).

### Sequence analysis to identify mutations.

Genomic DNA was extracted from 20 5-FOA-resistant mutants and 20 5-FU-resistant mutants isolated during fluctuation analysis of the wild-type, *msh2*Δ, *mlh1*Δ, and *pms1*Δ strains using a CTAB buffer (100 mM Tris-HCl [pH 7.5], 0.7 M NaCl, 10 mM EDTA, 1% β-mercaptoethanol, 1% CTAB [cetyltrimethylammonium bromide; Sigma-Aldrich]). The *URA5* gene was PCR amplified from 5-FOA-resistant mutants using primers KB187 and KB188 (Table S1). *FUR1* was PCR amplified from 5-FU-resistant mutants using primers KB207 and KB208. The PCR products were sequenced with the primers used for amplification and with an additional internal primer for *FUR1*, KB209 (Table S1). Sanger sequencing was performed at the Australian Genome Research Facility (AGRF).

Next-generation (Ion Torrent) sequencing was performed by the Australian Genome Research Facility. Totals of 16,381,698 and 16,742,455 reads were received for strains C23 and C45, respectively. The C23 and C45 sequences had a mean length of 100 bp, with 16.3 and 16.7 million reads, respectively (~85× coverage). Reads were aligned to the *C. neoformans* H99 genome sequence obtained from the JGI MycoCosm Fungal Genomics Resource (http://genome.jgi.doe.gov/Cryne_H99_1/Cryne_H99_1.home.html) using Geneious Pro. SNPs and sequence variations were identified within the genome using Geneious Pro with a variant *P* value of 10^−8^ (representing a 0.000001% likelihood of observation by chance), a minimum coverage rate of 50 reads, and a variant frequency rate at least 70%.

### Testing for sensitivity to or tolerance of DNA-damaging agents.

Strains were cultured overnight in YPD medium, 10-fold serially diluted, and plated onto YPD medium with or without the following stress agents: 0.04% methyl methanesulfonate (MMS) (Sigma-Aldrich; catalog no. 129925), 0.06% ethidium bromide solution (EtBr) (Sigma-Aldrich; catalog no. E1510), 0.25 mM menadione (Sigma-Aldrich; catalog no. M5625), 0.25 mM paraquat dichloride hydrate (paraquat) (Sigma-Aldrich; catalog no. 36541), 0.4 mM Luperox TBH70X tert-butyl hydroperoxide solution (tBOOH) (Sigma-Aldrich; catalog no. 458139), and 5 mM hydrogen peroxide solution (H_2_O_2_) (Sigma-Aldrich; catalog no. 216763). One set of plates was exposed to UV light (120 J m^−2^). Plates were incubated at 28°C for 2 days.

### Phenotypic characterization of microevolving strains.

The wild-type strain and the *msh2*Δ, *mlh1*Δ, and *pms1*Δ mutants were streaked for single colonies on YPD medium or YPD medium-nourseothricin (100 µg/ml) plates and incubated at 28°C. Single colonies were restreaked every 7 days for 2 months (approximately 600 generations). Three independent strains were generated for the wild-type strain and each mutant (Table S2). Strains were then cultured overnight using YPD medium, 10-fold serially diluted, and plated onto YPD medium with or without the following stress agents: 0.25 mM paraquat dichloride hydrate (paraquat) (Sigma-Aldrich; catalog no. 36541), 5 mM hydrogen peroxide solution (H_2_O_2_) (Sigma-Aldrich; catalog no. 216763), 1.5 mg/ml calcofluor white, and 5 mg/ml Congo red. Plates were incubated at 28°C for 2 days. One set of plates was incubated at 37°C. Strains were also plated on l-DOPA (l-3,4-dihydroxyphenylalanine) medium to assess melanization.

### Antifungal resistance.

Fluconazole Etests were performed on yeast nitrogen base agar plates (pH 7.0) per the manufacturer’s instructions and the method described in reference 40. To assess the frequency of colony formation on fluconazole or amphotericin B, 1 × 10^5^ cells of each strain from an overnight YPD culture were used to inoculate 3 separate YPD cultures. After growth for 48 h in a roller drum at room temperature, 1 × 10^6^ cells were plated onto YPD medium plus 72 µg/ml fluconazole (16× MIC) or 4.8 µg/ml amphotericin B (32× MIC) and the plates were incubated at 30°C for 7 days. Frequencies were calculated as the number of colonies on the drug plate divided by the total count of CFU plated. Frequency averages and standard errors of the mean were calculated using Prism 4.0c. Two-tailed Student’s *t* tests were performed to determine statistical significance.

### Virulence studies *in vivo* and fungal burden in infected organs.

Yeast strains were grown at 30°C overnight, and cultures were washed twice with phosphate-buffered saline (PBS) and resuspended at a final concentration of 2 × 10^5^ cells/ml. Groups of female A/Jcr mice (Jackson Laboratory, Bar Harbor, ME) were infected intranasally with 1 × 10^4^ yeast cells of each strain. Over the course of experiments, animals that appeared moribund or in pain were sacrificed by CO_2_ inhalation. Survival data from the murine experiments were statistically analyzed between paired groups by using the log rank test in Prism program 4.0 (GraphPad Software, Inc., San Diego, CA). *P* values of <0.01 were considered significant.

To compare fungal burdens, infected lungs and brains at the endpoint of the experiment were isolated and homogenized in PBS buffer using a homogenizer. Resuspensions were diluted, and 100 μl of each dilution was spread on YPD medium. Fungal colonies were counted after 3 days of incubation at 30°C. All statistical analyses were undertaken using the two-tailed Student’s *t* test. *P* values of <0.05 were considered statistically significant.

### Ethics statement.

This study was performed according to the guidelines of NIH and Institutional Animal Care and Use Committee (IACUC). The animal models and procedures used have been approved by the IACUC at Rutgers University (protocol 15041D0518).
