# Supplementary material for: Mismatch Repair of DNA Replication Errors Contributes to Microevolution in the Pathogenic Fungus Cryptococcus neoformans
Source: mBio. 2017 May 30;8(3):e00595-17. doi: 10.1128/mBio.00595-17 (PMC5449657; doi:10.1128/mBio.00595-17)
Supplement: TABLE S1 [file mbo003173331st1.doc]

| **Primer Name** | **Gene** | **Sequence** |
| --- | --- | --- |
| ALID2273 | *MSH2* | GGATCCACCAACTACCTTCTTGCAC |
| ALID2274 | *MSH2* | GGATCCAGAATTTTCCAAAGACGAGG |
| KB001 | *MLH1* | TCTAGAAAGAGAGAGCTGAGCTGC |
| KB002 | *MLH1* | AGGCCTCGGGATTGGGTGCGTTGG |
| ALID2277 | *PMS1* | ACTAGTTTTGAAGTAACCAGCTTCC |
| ALID2278 | *PMS1* | ACTAGTCATCATGCGGATAAGATCAG |
| KB012 | *MSH1* | AAGAGCTCACGACAACACCAAATCCC |
| KB013 | *MSH1* | ATGAGCTCTGGCTTCAGGCTGGTTAC |
| KB150 | *MSH1* | CAAGAGTTCCTGTTCCTG |
| KB151 | *MSH1* | ACTCGTAACCCTTCAATC |
| KB020 | *MSH3* | AACTCGAGGTTCTCTTTCCCGCCTGG |
| KB021 | *MSH3* | TTCTCGAGTTCCCTCTCCGTGCATTC |
| KB133 | *MSH3* | TTCTGTCTAGCGTCGATG |
| KB134 | *MSH3* | GTAGTGTATTTCGCTCC |
| KB028 | *MSH4* | ATGTCGACTGGCAGATGGTATTTCC |
| KB029 | *MSH4* | AAGGATCCGTGCTGAGGCGCTGAGTG |
| KB152 | *MSH4* | ATTCTGGTTGTATGATTG |
| KB153 | *MSH4* | CAATCATCTCATCAAAAT |
| KB036 | *MSH5* | AAACTAGTATATCGTGATTGCAGGGC |
| KB037 | *MSH5* | AAGGTACCCGAGGATAATGCGAGGTG |
| KB154 | *MSH5* | ATATTGAATGAAGCGTAC |
| KB155 | *MSH5* | CAGATGACATGCAATACC |
| KB187 | *URA5* | ACCGTGCCCGATAACATCTG |
| KB188 | *URA5* | TAGCCTCCTTTGTCGCTTC |
| KB207 | *FUR1* | CAAGGCACACAGCCATGA |
| KB208 | *FUR1* | CCATCGTCCGGCTGTACT |
| KB209 | *FUR1* | CAGTGCGAATTGGGAAGG |
