# Supplementary material for: Mismatch Repair of DNA Replication Errors Contributes to Microevolution in the Pathogenic Fungus Cryptococcus neoformans
Source: mBio. 2017 May 30;8(3):e00595-17. doi: 10.1128/mBio.00595-17 (PMC5449657; doi:10.1128/mBio.00595-17)
Supplement: TABLE S2 [file mbo003173331st2.doc]

| **Strain Name** | **Strain obtained/derived from** | **Genotype** |
| --- | --- | --- |
| KN99α | (41) | Wild type |
| AISVCN195 | (13) | *msh2*∆*::NAT* |
| KBCN008 | AISVCN195 | *msh2*∆*::NAT* [*MSH2 NEO*] |
| AISVCN196 | (13) | *mlh1*∆*::NAT* |
| KBCN0013 | AISVCN196 | *mlh1*∆*::NAT* [*MLH1 NEO*] |
| AISVCN198 | (13) | *pms1*∆*::NAT* |
| KBCN0021 | AISVCN198 | *pms1*∆*::NAT* [*PMS1 NEO*] |
| AISVCN202 | AISVCN195 x AISVCN198 | *msh2*∆*::NAT* *pms1*∆*::NAT* |
| AISVCN204 | AISVCN196 x AISVCN198 | *mlh1*∆*::NAT* *pms1*∆*::NAT* |
| AISVCN206 | AISVCN195 x AISVCN196 | *msh2*∆*::NAT* *mlh1*∆*::NAT* |
| KBCN0029 | KN99α | *msh1*∆*::NAT* |
| KBCN0031 | KN99α | *msh3*∆*::NAT* |
| KBCN0045 | KN99α | *msh4*∆*::NAT* |
| KBCN0035 | KN99α | *msh5*∆*::NAT* |
| KBCN0062 | KN99α  passaged 600 generations | Wild type |
| KBCN0063 | KN99α  passaged 600 generations | Wild type |
| KBCN0064 | KN99α  passaged 600 generations | Wild type |
| KBCN0065 | AISVCN195 passaged 600 generations | *msh2*∆*::NAT* |
| KBCN0066 | AISVCN195 passaged 600 generations | *msh2*∆*::NAT* |
| KBCN0067 | AISVCN195 passaged 600 generations | *msh2*∆*::NAT* |
| KBCN0068 | AISVCN196 passaged 600 generations | *mlh1*∆*::NAT* |
| KBCN0069 | AISVCN196 passaged 600 generations | *mlh1*∆*::NAT* |
| KBCN0070 | AISVCN196 passaged 600 generations | *mlh1*∆*::NAT* |
| KBCN0071 | AISVCN198 passaged 600 generations | *pms1*∆*::NAT* |
| KBCN0072 | AISVCN198 passaged 600 generations | *pms1*∆*::NAT* |
| KBCN0073 | AISVCN198 passaged 600 generations | *pms1*∆*::NAT* |
| C23 | (22) | Wild type |
| C45 | (22) | Wild type |
